# Prognostic Value and Efficacy Evaluation of Novel Drugs for Multiple Myeloma Patients with 1q21 Amplification (Amp1q21) Only: A Systematic Review of Randomized Controlled Trials

**DOI:** 10.7150/jca.40711

**Published:** 2020-02-19

**Authors:** Liang Chen, Zhendong Li, Shanshan Li, Weijun Fu, Rong Li

**Affiliations:** Department of Hematology, Navy Medical Center of PLA, Shanghai, China

**Keywords:** multiple myeloma, 1q21 amplification, therapy strategy, novel agents, systematic review

## Abstract

**Background:** Multiple myeloma (MM) is a heterogeneous disease characterized by chromosomal translocation, deletion, and amplification in plasma cells, resulting in a huge heterogeneity in its outcomes. Of all these cytogenetic abnormalities, Amp1q21 is most commonly detected, which is always associated with significantly shorter progression-free survival (PFS) and overall survival (OS) than normal 1q copy number status. In the era of novel agents such as bortezomib, ixazomib, lenalidomide, a head-to-head comparison of all these agents is still absent, especially in the patients with Amp1q21 alone. So, aiming to explore the optimum therapy to the patients with Amp1q21 only, we conduct this study.

**Patients and Methods:** We searched the PubMed, the Cochrane Library, PMC and the Embase databases, and we selected all the randomized controlled trials (RCTs) in English about MM with Amp1q21 up to April, 2019. A total of 72 papers were full screened and finally 2 literatures can be included in our study.

**Results:** Of the two studies, the one is about IRd (ixazomib, lenalidomide, dexamethasone) vs. placebo-Rd (HR, 0.781; 95% CI, 0.492-1.240), another is about VAD (vincristine, adriamycin, dexamethasone) vs. PAD (bortezomib, adriamycin, dexamethasone) (3-year survival rate: 59% vs. 83%, p=0.016).

**Conclusion:** From this review, MM patients with Amp1q21 may somewhat benefit from ixazomib but the evidence is still stuffless. What's more, a head-to-head comparison between ixazomib and other agents among MM patients with Amp1q21 is also absent. So, we sincerely expect this review can attract some attention for the therapy of this special part of patients. This study was registered in https://www.crd.york.ac.uk/prospero/#recordDetails.

## Introduction

MM accounts for 1% of all cancers and approximately 10% of all hematologic malignancies 1, 2. In China, the incidence of multiple myeloma is about 10-20 / million / per years, which ranks the second in the incidence of hematological malignant tumors. And MM is always newly diagnosed aging 65 to 74, with a median age of 69 3.

MM is more recently being recognized as a heterogeneous group of disease characterized by chromosomal translocation, deletion, and amplification in plasma cells, resulting in a huge heterogeneity in its outcomes 4. Above all these chromosomal abnormalities, Amp1q21, t (4; 14), 17p-, t (14; 16), t (14; 20) were with a prevalence of 33%, 12%-15%, 6.6%- 11%, 3%-5%, 1.5%-3%, respectively 5,6. Obviously, Amp1q21 is the most common cytogenetic abnormality. What's more, Amp1q21 is always means a poor prognostic and Amp1q21 is as an independent adverse prognostic factor 7. In 2012, the Amp1q21 was as a high risk factor according to a prognostic classification system 8; in 2015, patients with t (4; 14) translocation and gain (1q) were classified into intermediate-risk group 6, 9; and in 2018, updated mayo Stratification of Myeloma and Risk-Adapted Therapy (mSMART) combined the middle-risk and high-risk groups, and Amp1q21 was again as a poor- prognostic factor for multiple myeloma. At the same time, the concept of “double-hit” and “triple-hit” was proposed (defined as that: 2 or even 3 high-risk genetic abnormalities existence the same time.)^10^, ^11^. Also, amount of clinic trials had confirmed its prognostic outcomes. A retrospective analysis of 229 patients conducted in Beijing, China showed that Amp1q21 could be seen as an adverse prognostic factor for PFS (1q21 gain vs. non-1q21 gain: 25.0 vs. 36.0 months, P < 0.001) 12. Another study explains that MM patients in standard risk have a median overall survival (OS) of more than 7 years while those in high risk have a median OS of approximately 3 years despite tandem autologous stem cell transplantation (ASCT) 6. And a study of 500 patients showed that NDMM with Amp1q21 had inferior 5-year event-free/overall survival compared with those non-Amp1q21 (38%/52% vs. 62%/78%, both P < .001), and relapsed patients who had Amp1q21 had inferior 5-year post-relapse survival compared with those lacking Amp1q21 at relapse (15% vs. 53%, P = .027) 13. Besides, Amp1q21 as the secondary genetic events may contributes much to the relapse of myeloma, while the relapse rate may decrease if treating as early as possible 14, suggesting that an early treatment target on Amp1q21 in newly diagnosed MM (NDMM) may significantly decrease the relapse of MM patients.

So far, a steady flow of approved therapeutic agents have come to the fore for MM patients with cytogenetic abnormalities recently or in the projected further. A group of studies have showed that bortezomib can improve complete response, progression-free survival (PFS), and overall survival (OS) in t (4; 14) and Del (17/17p) 15, 16. And for the patients with Amp1q21, there are also some studies proved that the bortezomib and ixazomib may be effective, but the evidence is still not sufficient 17-19. In this study, we will integrate all available evidence, describe the statement of the treatment for MM patients with Amp1q21 and compare all the regimens in this study to explore an optimum therapy for these patients.

## Patients and Methods

### Search strategy

We searched PubMed, the Corcoran Library, PMC and the Embase used the terms "multiple myeloma" and "1q21" and all the agents, such as "daratumumab" ixazomib "panobinostat" and so on. All RCTs in English up to April 2019 were included. See Appendix 1 for further detail of retrieval.

### Study selection

Inclusion criteria:(1) Adult multiple myeloma patients with 1q21 amplification only; (2) RCTs; (3) the hypotheses and methods of each study are similar; (4) there is a date of the study be carried out or published; (5) the sample size of each study is clearly defined; (6) there are clear criteria for the selection of patients and the diagnosis and staging of cases in each study. (7) if the study report can provide OR (RR, rate difference, HR) and its 95% confidence interval, or can be transformed into OR (RR, rate difference, HR) and its 95% confidence interval; in the case of measurement information, mean, standard deviation and sample size should be provided (8) full articles with English language.

Exclusion criteria: (1) Multiple myeloma patients with t(4:14), t(14:16), p53- or other cytogenetic abnormalities though with 1q21 amplification; (2) repeated reports; (3) there are defects in research design and poor quality; (4) incomplete data and unclear outcome effect; (5) the statistical method is wrong and cannot be corrected and cannot be provided or can be converted into OR (RR, rate difference, HR) and its 95% confidence interval, Measurement information does not provide averages and standard deviations. Study selection will be carried out by 2 investigators independently, third investigators will participant if there are some differences.

### Study quality appraisal

Study quality was also independently assessed by 2 investigators according to the Corcoran collaboration Network bias risk Assessment tool. This tool mainly includes 6 dimensions: random sequence generation; assignment hiding; blinding; incomplete outcome data; selective reporting of outcome; others. We assessed the validity of data extraction by comparing the independently abstracted results for concordance, and any discrepancies were resolved by discussion and review of the original manuscript by the 2 investigators who extracted the data or, if necessary, a committee comprising all the investigators.

### Data extraction

For each of the articles satisfying the inclusion criteria, data were independently extracted by 2 investigators, which were subsequently examined by other authors to settle discrepancies. The following information was obtained from each publication: trial details (e.g., trial or author name, treatment details, number of participants, publication source), survival outcomes, and response rates. Survival outcomes included PFS, TTP, and OS and their corresponding CIs; both medians and HRs were recorded. The HR and 95% CI were directly determined from the articles. Furthermore, we attempted to identify the potentially relevant studies by tracing the reference list of pertinent manuscripts as well as contacting known authors in the articles.

## Results

### Systematic literature review

**Figure 1:**
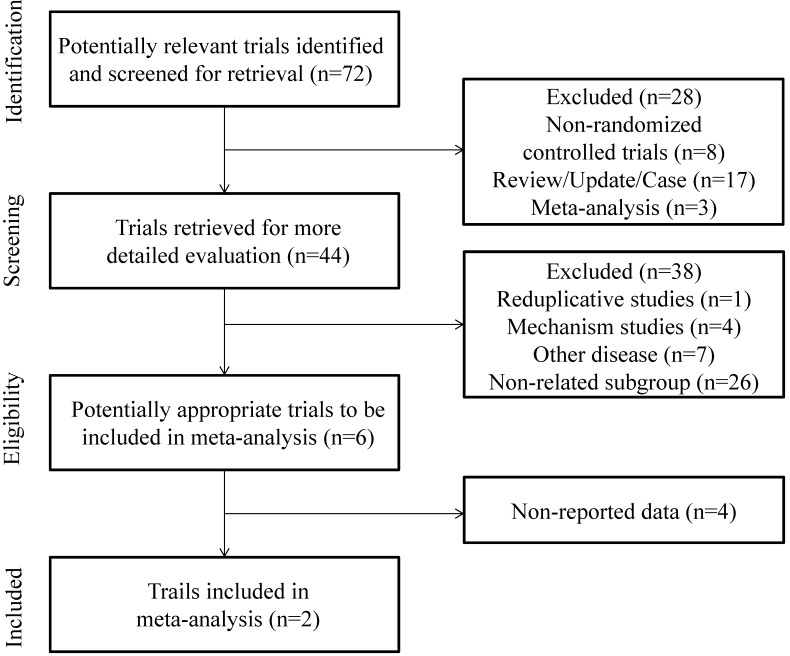
PRISMA 2009 flow diagram 30: MM patients with 1q21 amplification Phase III randomized.

Figure 1 presents the PRISMA flow diagram. This systematic literature review (SLR) identified a total of 72 citations from the databases. Based on the title and abstract, 28 citations were excluded because of non-randomized controlled studies, review, update, case reports and meta-analysis. The full text of remaining 44 citations were reviewed, and 38 studies were excluded because there were 4 about mechanism studies, 7 about other disease, and there existed the same trial, and twenty-six citations did not refer to the subgroup of 1q21 amplification only. In the second full text review of the remaining 6 citations, 4 didn't refer to the related data. Finally, the remaining 2 citations were included in our study after a complete assessment.

**Figure 2:**
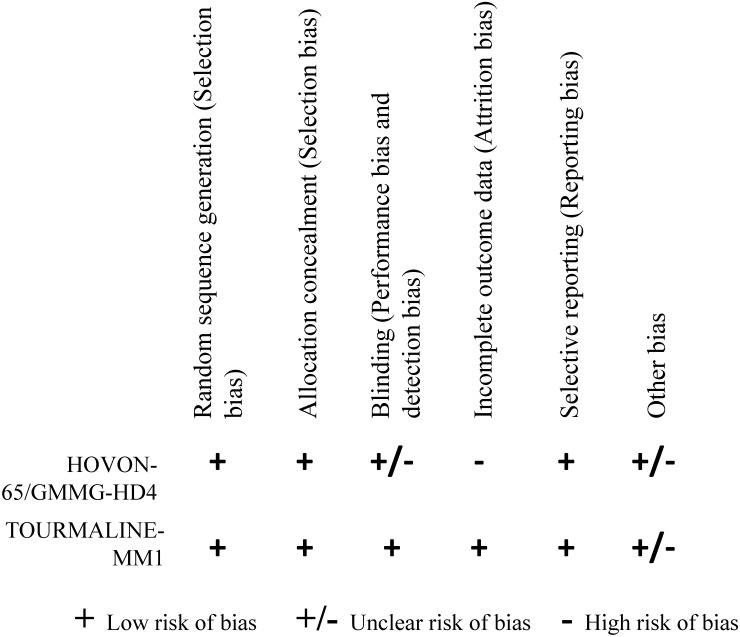
Methodological quality of the included studies.

2 RCTs were finally included in our study after assessed according to the Corcoran collaboration Network bias risk Assessment tool. The 2 RCTs included 4 treatment options: 1) vincristine, adriamycin, dexamethasone (VAD), 2) bortezomib, adriamycin, dexamethasone (PAD), 3) ixazomib, lenalidomide, dexamethasone (IRd), 4) lenalidomide, dexamethasone (placebo-Rd). Figure 2 showed detail of the methodological quality of the included studies.

### Data extraction and citations presentation

Two studies were included in this review and the details showed in Table 1. The one was conducted by Hartmut Goldschmidt et al. in 2010, which evaluated the association of FISH results and outcome of a subgroup of patients within the HOVON-65/ GMMG-HD4 trial, a prospective, randomized phase III trial for patients with NDMM stage II or III according to Salmon & Durie up to 65 years. 626 patients were consecutively enrolled in their study and they were randomized to receive three cycles of VAD (arm A; vincristine, adriamycin, dexamethasone) or PAD (arm B; bortezomib, adriamycin, dexamethasone). All the enrolled 626 patients were analyzed and 284 patients was included in their study. Of all the 284 patients, 258 patients' FISH results were available (n=131 in arm A; n=127 in arm B). Of all the FISH-available patients, 87 were detected with 1q21 gain (33.7 %, A: 33.1% vs. B: 34.4%). When comparing patients in the two arms for PFS and OS, median PFS for patients with gain 1q21 was 22 months (arm A) vs. 30 months (arm B) compared to 41 months in both arms for patients without gain 1q21. Patients with gain 1q21 showed a significantly better OS when treated with PAD (3yr-OS rates: A: 59%, B: 83%, p=0.016) 19.

Another study was launched in Blood in 14 DECEMBER, 2017 by Herve´ Avet-Loiseau et al. In their paper, the global, randomized, double-blind, placebo-controlled, phase 3 TOURMALINE-MM1 study enrolled a total of 722 patients from 147 sites in 26 countries between 28 August 2012 and 27 May 2014. Of all the enrolled patients, 172 were detected with Amp1q21 alone (IRd, n= 80; placebo-Rd, n= 92). The HR for all the 172 patients' PFS was 0.781 (95%CI, 0.492-1.240, p = 0.293) in favor of IRd and the medians were 15.4 months vs. 11.3 months in the IRd vs. placebo-Rd groups, respectively. And in this study, we can see that in patients with isolated Amp1q21, a improvement of PFS benefit from IRd was obvious in 20% and 60% cutoffs (HR: 0.682 and 0.683, respectively), and showed a poor results in 3% cutoff (HR: 0.781; 95% CI: 0.492-1.240) 17.

## Discussion

Amp1q21 is an independent poor-prognostic mark in MM patients. Of all the cytogenetic abnormality, Amp1q21 is the most common cytogenetic abnormality with a detection rate of 30%-35%. And Amp1q21 are always detected with other cytogenetic abnormalities. The worst thing is that MM with Amp1q21 is easier to relapse. So, an optimum therapy for MM patients with Amp1q21 may not only improve the survival of these patients, but reduce the recurrence to some extent.

Nowadays, amount of studies about Amp1q21 sprung up. As for the treatment for MM patients with cytogenetic abnormalities, proteasome inhibitor is still as the main choice. Of the two papers, the first is about the bortezomib, which proved that the clinical outcome of patients with 1q21 gain can be improved in those who received no less than 4 cycles of bortezomib-based therapy (bortezomib, thalidomide, and dexamethasone). Of course, there are some studies pointed that bortezomib cannot improve the prognostic of the MM patients with Amp1q21, but shows a better PFS or OS in treat of the patients with t(4; 14) or del(17/17p) 15, 16. Ixazomib, as the second generation proteasome inhibitor, shows an advantageous efficiency in the treatment of patients with Amp1q21. However, the prognostic value for Amp1q21 of the novel therapies is still need to be confirmed because the relevant study is so few that the evidence is not that credible. What's more, there is still vacancy in the comparison between the bortezomib and ixazomib, especially for the MM patients with Amp1q21.

Except for above two RCTs, there are also some other studies reported the therapy for MM patients with Amp1q21. Gang An, 20, 21 et al. and Fei Li, [22]et al. suggested that MM patients with chromosome 1q21 aberrations couldn't benefit significantly from bortezomib-based regimes, which showed a not superior efficiency than thalidomide. Among the regimes incorporating bortezomib, Mai E K, [23]et al. conducted a study to compare the bortezomib, cyclophosphamide and dexamethasone (VCD) versus bortezomib, doxorubicin and dexamethasone (PAd) in NDMM patients and observed that objects with chromosome 1q21 aberrations was more likely to develop a progressive disease when treated with PAd than CBD. Apart from the proteasome inhibitor, elotuzumab was also observed beneficial for patients with high-risk cytogenetic abnormalities including del(17p), 1q21 gain/amplification, and particularly t(4;14) by Lonial S, et al. in the ELOQUENT-2 study 24. Besides, pomalidomide was proved safe and effective in RRMM with high-risk cytogenetic features including Amp1q21 25. Though all above studies mentioned the efficiency of agents on MM patients with Amp1q21, but little data was reported just as the Meta analysis conducted in 2016 26. From our searching results, almost all the studies referred to the Amp1q21 but stop on the point, which may indicated the importance of Amp1q21, but for such an important cytogenetic abnormality, we need more data rather than a simple affirmation.

Almost all MM patients relapse after induction therapy, but the relapse mechanisms are still in shadowy. In a study conducted by Florence Magrangeas et al., a hypothesis of 'subclones evolution' was proposed just as natural selection by Darwin, and the launcher attributed the problem to the minor subclone, which can survive and expand while the dominant subclone is killed by therapy, providing a reservoir for relapse. This pattern can be observed preferentially in patients treated with bortezomib, though bortezomib shows a more preferable outcome than conventional induction chemotherapy. Furthermore, 1q21 gain or Amp1q21, as the secondary genetic event, was investigated and found that relapse was associated with a significant increase to 19.1 in the mean number of CNAs per case when patients with 1q21 gain, NF-κB activating mutations and TP53 deletion (P < 0.001) 27. Despite the evidence provided by above study, we also found the direct evidence that detection rate in relapse MM patients is higher than that in NDMM patients all the time 5, 21, 28. In an early study, the stem cell marker nestin strongly associated with the presence of 1q21 gain may play an important role in MM relapse 29. Besides, a study in Beijing analysis a gene expression microarrays of 1878 MM patients and found that with the increase of the amplification level of 1q21, the expression level of BCAR3 which is a protein-coding gene associated with many tumors showed an overall downward trend, interestingly, the expression of BCAR3 gene was different statistically in the non-relapse group and the relapse group, and the expression in the non-relapse group was notably higher than that in the relapse group (P = 0.0023) 14. In a word, a common view have get that the main cause of myeloma recurrence is persistent residual tumor cells, which are associated with clonal evolution and immune dysfunction. Amp1q21 as the secondary genetic events may contributes much to the relapse of myeloma. So an effective therapy to the MM patients with Amp1q21 may overcome the relapse partially. We certainly expect a deep-going study about the detailed relationship between relapse mechanisms and Amp1q21.

From this review, we can get that: 1) Proteasome inhibitor is still as the first choice to the patients with cytogenetic abnormality for the moment, MM patients with Amp1q21 isolate may benefit from IRd and the improvement may somewhat depend on the clone size of Amp1q21, but its efficacy is still to be confirmed by amounts of RCTs. 2) As the most frequency cytogenetic abnormality, Amp1q21 should get much attention of us and we expect that there will be numerous studies arising. 3) Risk stratification, which is based on a wealth of data from epidemiological studies and clinical trials, combined with patient clinical manifestations and laboratory findings, has become an important way to select the advantageous therapy for patients and we believe that, in the near future, the therapy will vary from patient to patient according to the risk stratification to achieve the precise hit. Of course, there are also some limitations, for instance, the included study is so less that the evidence is not so sufficient, which may also deliver us that: 1) there is still a spectacular space in the treatment of MM patients, especially with Amp1q21; 2) a comparison of the efficiency of novel agents is absent, and especially for the MM patients with various cytogenetic abnormalities; 3) whether ixazomib could improve the adverse prognosis, either in patients with Amp1q21 only or with other cytogenetic abnormalities, and what about the degree of improvement is still a puzzle; 4) a further and precise study of the mechanisms between the Amp1q21 and relapse, drug resistance and the response to novel agents is expected.

## Supplementary Material

Online Appendix 1 Search strategies.

## Figures and Tables

**Table 1:** Trial Details and Patient Characteristics.

No	Trial Name/First Author	Research			Control			HR	p value	disease status	year	Median Age, years (range )	Primary Objective
		Arm A	Num	PFS/OS	Arm B	Num	PFS/OS						
**1**	HOVON-65/GMMG-HD4 /Hartmut Goldschmidt	VAD	43	22mo/ 3y(OS) 59%	PAD	44	30mo/ 3y(OS) 83%	NR	0.016 (OS)	NDMM	2010	<65	PFS /OS
**2**	TOURMALINE-MM1 study/ Herve´ Avet-Loiseau	IRd	80	15.4mo /16.4mo	placebo-Rd	92	11.3mo /12.3mo	0.781 (0.492-1.240)	0.293	RRMM	14 DEC 2017	67.0 (45-86)	PFS /TTP

Abbreviation: Num: the sample size of each group; PFS: progression-free survival; OS: overall survival; HR: hazard ratio; VAD: vincristine, adriamycin, dexamethasone; PADL: bortezomib, adriamycin, dexamethasone; mo: months; y: years; NR: no report; NDMM: newly diagnosed multiple myeloma; IRd: ixazomib, lenalidomide, dexamethasone; placebo-Rd: placebo, lenalidomide, dexamethasone; RRMM: relapse and refractory multiple myeloma; TTP: time to progression.
