# Supplementary material for: Prognostic Value and Efficacy Evaluation of Novel Drugs for Multiple Myeloma Patients with 1q21 Amplification (Amp1q21) Only: A Systematic Review of Randomized Controlled Trials
Source: J Cancer. 2020 Feb 19;11(9):2639–44. doi: 10.7150/jca.40711 (PMC7066010; doi:10.7150/jca.40711)
Supplement: Supplementary file 1 — Online Appendix 1 Search strategies. [file jcav11p2639s1.pdf]

Prognostic value and efficacy evaluation of novel drugs for multiple myeloma patients with 1q21 amplification only: A systematic review of randomized controlled trials

Liang Chen, Rong Li, et al.

Online Appendix 1 Search strategies

1.1 Embase

Database name	Embase
Search interface	http://www.embase.com
Date of search	8 April 2019
Time segment	2010 to 2019
Search filter	-

Table 1: Embase search strategy for randomized controlled trials

#	Search term
1	'multiple myeloma'/de
2	'myeloma'/de
3	'myeloma cell'/de
4	myelom*
5	#1 OR #2 OR #3 OR #4
6	'bortezomib'/de
7	bortezomib:ab,ti OR velcade:ab,ti OR ps341:ab,ti OR 'ps-341':ab,ti OR (ps NEAR/1 '341'):ab,ti OR (proteasome NEXT/1 inhibit*):ab,ti
8	'lenalidomide'/de
9	lenalidomide:ab,ti OR revimid:ab,ti OR revlimid:ab,ti OR 'cc 5013':ab,ti OR cc5013:ab,ti OR 'cdc 501':ab,ti OR 'cdc 5013':ab,ti OR cdc501:ab,ti OR cdc5013:ab,ti OR 'enmd 0997':ab,ti OR enmd0997:ab,ti OR 'imid 3':ab,ti OR imid3:ab,ti
10	'thalidomide'/de
11	thalidomide:ab,ti OR thalidomid:ab,ti OR thalimodide:ab,ti OR thalomid:ab,ti OR contergan:ab,ti OR distaval:ab,ti OR isomin:ab,ti OR 'k-17':ab,ti OR kedavon:ab,ti OR kevadon:ab,ti OR neurosedin:ab,ti OR neurosedyne:ab,ti OR 'nsc 66847':ab,ti OR sedalis:ab,ti OR 'shin naito':ab,ti OR softenon:ab,ti OR synovir:ab,ti OR talimol:ab,ti OR talizer:ab,ti OR telagan:ab,ti OR telargan:ab,ti
12	'bendamustine'/de
13	bendamustine:ab,ti OR 'cimet 3393':ab,ti OR cytostasan:ab,ti OR cytostasane:ab,ti OR 'imet 3393':ab,ti OR ribomustin:ab,ti OR treanda:ab,ti
14	'dexamethasone'/de

#	Search term
15	'aeroseb dex':ab,ti OR aflucoson*:ab,ti OR anaflogistico:ab,ti OR arcodexan*:ab,ti OR azium:ab,ti OR calonat:ab,ti OR cebedex:ab,ti OR colofoam:ab,ti OR cortidron*:ab,ti OR cortisumman:ab,ti OR dacortin*:ab,ti OR dalalone:ab,ti OR decacortin:ab,ti OR decadeltoson*:ab,ti OR decadion:ab,ti OR decadr*n*:ab,ti OR decaesadril:ab,ti OR decamethasone:ab,ti OR decasone:ab,ti OR decaspray:ab,ti OR decasterolone:ab,ti OR decilone:ab,ti OR decofluor:ab,ti OR dectancyl:ab,ti OR dekacort:ab,ti OR delladec:ab,ti OR deltafluoren:ab,ti OR deltafluorene:ab,ti OR dergramin:ab,ti OR deronil:ab,ti OR desacort:ab,ti OR desacortone:ab,ti OR desadrene:ab,ti OR desalark:ab,ti OR desameton*:ab,ti OR 'dexa cortisyl':ab,ti OR 'dexa dabrosan':ab,ti OR 'dexa korti':ab,ti OR 'dexa scherosan':ab,ti OR 'dexa scherozon':ab,ti OR 'dexa scherozone':ab,ti OR dexachel:ab,ti OR dexacort*:ab,ti OR dexadabroson:ab,ti OR dexadecadrol:ab,ti OR dexadrol:ab,ti OR dexagen:ab,ti OR dexahelvacort:ab,ti OR dexakorti:ab,ti OR dexalocal:ab,ti OR dexamecortin:ab,ti OR dexameson*:ab,ti OR dexametason*:ab,ti OR dexameth:ab,ti OR dexametha*on*:ab,ti OR dexamethonium:ab,ti OR dexan:ab,ti OR dexane:ab,ti OR dexapot:ab,ti OR dexaschero*on*:ab,ti OR dexason*:ab,ti OR dexinoral:ab,ti OR dexionil:ab,ti OR dexone:ab,ti OR dextelan:ab,ti OR dezone:ab,ti OR doxamethasone:ab,ti OR esacortene:ab,ti OR exadion*:ab,ti OR firmalone:ab,ti OR fluormone:ab,ti OR fluorocort:ab,ti OR fluorodelta:ab,ti OR fortecortin:ab,ti OR gammacorten*:ab,ti OR grosodexon*:ab,ti OR hexadecad*ol:ab,ti OR hexadiol:ab,ti OR hexadrol:ab,ti OR isnacort:ab,ti OR isoptodex:ab,ti OR isoptomaxidex:ab,ti OR 'lokalison f':ab,ti OR luxazone:ab,ti OR marvidione:ab,ti OR maxidex:ab,ti OR mediamethasone:ab,ti OR megacortin:ab,ti OR mephameson*:ab,ti OR metasolon*:ab,ti OR methazonion*:ab,ti OR millicorten:ab,ti OR millicortenol:ab,ti OR 'mk 125':ab,ti OR mk125:ab,ti OR nisomethasona:ab,ti OR novocort:ab,ti OR 'nsc 34521':ab,ti OR nsc34521:ab,ti OR opticorten:ab,ti OR opticortinol:ab,ti OR oradex*n*:ab,ti OR orgadrone:ab,ti OR policort:ab,ti OR posurdex:ab,ti OR prodexona:ab,ti OR prodexone:ab,ti OR sanamethasone:ab,ti OR spoloven:ab,ti OR triamcimetil:ab,ti OR visumethazone:ab,ti
16	'melphalan'/de
17	melph*lan:ab,ti OR alkeran:ab,ti OR 'cb 3025':ab,ti OR cb3025:ab,ti OR 'levo sarcolysin':ab,ti OR levofalan:ab,ti OR melfalan:ab,ti OR melphalon:ab,ti OR 'nsc 8806':ab,ti OR nsc8806:ab,ti OR 'phenylalanine 2037':ab,ti OR 'phenylalanine mustard':ab,ti
18	'vincristine'/de
19	vincristine:ab,ti OR vincristin:ab,ti OR 'l 37231':ab,ti OR l37231:ab,ti OR 'vin cristine':ab,ti OR vincrisul:ab,ti
20	'cyclophosphamide'/de
21	cyclophosphamide:ab,ti OR 'b 518':ab,ti OR b518:ab,ti OR carloxan:ab,ti OR clafen:ab,ti OR cycloblastin*:ab,ti OR 'cyclofos amide':ab,ti OR cyclofosfamid*:ab,ti OR cyclophosphamid*:ab,ti OR cyclophosphan*:ab,ti OR cyclostin:ab,ti OR cycloxan:ab,ti OR cyphos:ab,ti OR cytophosphan*:ab,ti OR cytoxan:ab,ti OR 'endocyclo phosphate':ab,ti OR end*xan*:ab,ti OR genoxal:ab,ti OR 'mitoxan neosan':ab,ti OR neosar:ab,ti OR noristan:ab,ti OR 'nsc 26271':ab,ti OR 'nsc 2671':ab,ti OR procytox:ab,ti OR procytoxide:ab,ti OR se*doxan:ab,ti
22	'doxorubicin'/de

#	Search term
23	doxorubicin:ab,ti OR adriablastin:ab,ti OR adriablastin*:ab,ti AND adriacin:ab,ti OR adriamicin*:ab,ti OR adriblastin*:ab,ti OR caelyx:ab,ti OR doxil:ab,ti OR doxorubicine:ab,ti OR 'fi 106':ab,ti OR fi106:ab,ti OR lipodox:ab,ti OR myocet:ab,ti OR 'nsc 123127':ab,ti OR nsc123127:ab,ti OR rastocin:ab,ti OR resmycin:ab,ti OR 'rp 25253':ab,ti OR rp25253:ab,ti OR rubex:ab,ti OR sarcodoxome:ab,ti OR 'tlc d 99':ab,ti
24	'carmustine'/de
25	carmustine:ab,ti OR bcnu:ab,ti OR bicnu:ab,ti OR carmubis:ab,ti OR carmubris:ab,ti OR carmustin:ab,ti OR gliadel:ab,ti OR nitrumon:ab,ti OR 'nsc 409962':ab,ti
26	'prednisone'/de
27	prednisone:ab,ti OR ancortone:ab,ti OR biocortone:ab,ti OR colisone:ab,ti OR cortidelt:ab,ti OR 'de cortisyl':ab,ti OR decortancyl:ab,ti OR de*ortin*:ab,ti OR dehydrocortisone:ab,ti OR delitisone:ab,ti OR deltacort*n*:ab,ti OR deltacortisone:ab,ti OR deltasone:ab,ti OR deltra:ab,ti OR 'di-adreson':ab,ti OR diadreson:ab,ti OR en*orton*:ab,ti OR hostacortin:ab,ti OR insone:ab,ti OR meprison:ab,ti OR metacortandracin:ab,ti OR meticorten:ab,ti OR meticortine:ab,ti OR 'nsc 10023':ab,ti OR nsc10023:ab,ti OR orasone*:ab,ti OR paracort:ab,ti OR precort:ab,ti OR precortal:ab,ti OR prednisone*:ab,ti OR pronizone:ab,ti OR rectodelt:ab,ti OR ultracorten:ab,ti OR urtilone:ab,ti
28	'prednisolone'/de
29	prednisolone:ab,ti OR antisolon*:ab,ti OR aprednislon*:ab,ti OR benisolon*:ab,ti OR berisolon*:ab,ti OR caberdelta:ab,ti OR 'co hydeltra':ab,ti OR codelcortone:ab,ti OR cortadelton*:ab,ti OR cortelinter:ab,ti OR cortisolone:ab,ti OR dacortin:ab,ti OR decortril:ab,ti OR dehydrocortex:ab,ti OR dehydrocortisol*:ab,ti OR dehydrohydrocortison*:ab,ti OR delcortol:ab,ti OR deltacortef:ab,ti OR deltacortenolo:ab,ti OR deltacortil:ab,ti OR deltacortoil:ab,ti OR deltaderm:ab,ti OR deltaglycortril:ab,ti OR deltahycortol:ab,ti OR deltahydrocortison*:ab,ti OR deltaophticor:ab,ti OR deltasolone:ab,ti OR deltastab:ab,ti OR deltidrosol:ab,ti OR deltisilone:ab,ti OR deltisolon*:ab,ti OR deltolasson*:ab,ti OR deltoson*:ab,ti OR dicortol:ab,ti OR domucortone:ab,ti OR encort*lon*:ab,ti OR glistelone:ab,ti OR hostacortin:ab,ti OR hydeltra:ab,ti OR hydeltrone:ab,ti OR hydrelta:ab,ti OR hydrocortancyl:ab,ti OR hydrocortidelt:ab,ti OR hydrodeltalone:ab,ti OR hydrodeltisone:ab,ti OR hydroretrocortin*:ab,ti OR inflanefran:ab,ti OR insolone:ab,ti OR keteocort:ab,ti OR leocortol:ab,ti OR mediasolone:ab,ti OR meprisolon*:ab,ti OR metacortalon*:ab,ti OR metacortandralon*:ab,ti OR metacortelone:ab,ti OR meticortelone:ab,ti OR metiderm:ab,ti OR morlone:ab,ti OR mydrapred:ab,ti OR nisolon:ab,ti OR nisolone:ab,ti OR 'nsc 9120':ab,ti OR nsc9120:ab,ti OR panafcortolone:ab,ti OR panafort:ab,ti OR paracortol:ab,ti OR phlogex:ab,ti OR precortalon:ab,ti OR precortancyl:ab,ti OR precortisyl:ab,ti OR predartrin*:ab,ti OR prednedome:ab,ti OR prednelan:ab,ti OR prednicoelin:ab,ti OR prednicort:ab,ti OR prednicortelone:ab,ti OR prednifor:ab,ti OR predniment:ab,ti OR predniretard:ab,ti OR prednis:ab,ti OR prednivet:ab,ti OR prednorsolon*:ab,ti OR predonine:ab,ti OR predorgasolon*:ab,ti OR prelone:ab,ti OR prenolone:ab,ti OR prezolon:ab,ti OR scherisolon:ab,ti OR serilone:ab,ti OR solone:ab,ti OR solupren*:ab,ti OR spiricort:ab,ti OR spolotane:ab,ti OR sterolone:ab,ti OR supercorti*ol:ab,ti OR taracortelone:ab,ti OR wysolone:ab,ti

#	Search term
30	‘pomalidomide’/de
31	pomalidomide:ab,ti OR imnovid:ab,ti OR pomalyst:ab,ti OR 'cc-4047':ab,ti OR 'cc 4047':ab,ti OR cc4047:ab,ti
32	‘panobinostat’/de
33	panobinostat:ab,ti OR farydak:ab,ti OR ‘lbh-589’:ab,ti OR ‘lbh589’:ab,ti OR ‘lbh 589’:ab,ti
34	‘carfilzomib’/de
35	carfilzomib:ab,ti OR kyprolis:ab,ti OR ‘pr-171’:ab,ti OR ‘pr171’:ab,ti OR ‘pr 171’:ab,ti
36	‘daratumumab’/de
37	daratumumab:ab,ti OR darzalex:ab,ti
38	‘ixazomib’/de
39	ixazomib:ab,ti OR ninlaro:ab,ti OR mln9708:ab,ti OR ‘mln 9708’:ab,ti OR ‘mln-9708’:ab,ti
40	‘elotuzumab’/de
41	elotuzumab:ab,ti OR empliciti:ab,ti OR HuLuc63:ab,ti OR BMS-901608:ab,ti
42	#6 OR #7 OR #8 OR #9 OR #10 OR #11 OR #12 OR #13 OR #14 OR #15 OR #16 OR #17 OR #18 OR #19 OR #20 OR #21 OR #22 OR #23 OR #24 OR #25 OR #26 OR #27 OR #28 OR #29 OR #30 OR #31 OR #32 OR #33 OR #34 OR #35 OR #36 OR #37 OR #38 OR #39 OR #40 OR #41
43	'randomization'/de
44	'controlled study'/de
45	'comparative study'/de
46	‘blind procedure'/de
47	'placebo'/de
48	'controlled clinical trial' OR 'controlled clinical trials'
49	'randomised controlled trial' OR 'randomized controlled trial' OR 'randomised controlled trials' OR 'randomized controlled trials'
50	rct
51	placebo*
52	#43 OR #44 OR #45 OR #46 OR #47 OR #48 OR #49 OR #50 OR #51
53	1q21
54	#5 AND #42 AND #52 AND #53

1.2 Cochrane

Database name Cochrane

Search interface http://www.thecochranelibrary.com

Date of search 8 April 2019

Time segment 2010 to 2019

Search filter Controlled clinical trials

#	Search term
1	myeloma*
2	MeSH descriptor: [Multiple Myeloma] explode all trees
3	proteasome inhibitor
4	bortezomib
5	(velcade OR ps341 OR "ps-341" OR (ps NEAR/1 341))
6	lenalidomide
7	revimid OR revlimid OR "cc 5013" OR cc5013 OR "cdc 501" OR "cdc 5013" OR cdc501 OR cdc5013 OR "enmd 0997" OR enmd0997 OR "imid 3" OR imid3
8	thalidomide
9	thalidomid OR thalimodide OR thalomid OR contergan OR distaval OR isomin OR "k-17" OR kedavon OR kevadon OR neurosedin OR neurosedyne OR "nsc 66847" OR sedalis OR "shin naito" OR softenon OR synovir OR talimol OR talizer OR telagan OR telargan
10	bendamustine
11	"cimet 3393" OR cytostasan OR cytostasane OR "imet 3393" OR ribomustin OR treanda
12	MeSH descriptor: [Dexamethasone] this term only
13	MeSH descriptor: [Thalidomide] this term only
14	MeSH descriptor: [Melphalan] this term only
15	MeSH descriptor: [Vincristine] this term only
16	MeSH descriptor: [Cyclophosphamide] this term only
17	MeSH descriptor: [Doxorubicin] this term only
18	MeSH descriptor: [Carmustine] this term only
19	MeSH descriptor: [Prednisone] this term only
20	MeSH descriptor: [Prednisolone] this term only

#	Search term
21	('aeroseb dex' OR aflucoson* OR anaflogistico OR arcodexan* OR azium OR calonat OR cebedex OR colofoam OR cortidron* OR cortisumman OR dacortin* OR dalalone OR decacortin OR decadeltoson* OR decadion OR decadr*n* OR decaesadril OR decamethasone OR decasone OR decaspray OR decasterolone OR decilone OR decofluor OR dectancyl OR dekacort OR delladec OR deltafluoren OR deltafluorene OR dergramin OR deronil OR desacort OR desacortone OR desadrene OR desalark OR desameton* OR 'dexa cortisyl' OR 'dexa dabrosan' OR 'dexa korti' OR 'dexa scherosan' OR 'dexa scherozon' OR 'dexa scherozone' OR dexachel OR dexacort* OR dexadabroson OR dexadecadrol OR dexadrol OR dexagen OR dexahelvacort OR dexakorti OR dexalocal OR dexamecortin OR dexameson* OR dexametason* OR dexameth OR dexametha*on* OR dexamethonium OR dexan OR dexane OR dexapot OR dexaschero*on* OR dexason* OR dexinoral OR dexionil OR dexone OR dextelan OR dezone OR doxamethasone OR esacortene OR exadion* OR firmalone OR fluormone OR fluorocort OR fluorodelta OR fortecortin OR gammacorten* OR grosodexon* OR hexadecad*ol OR hexadiol OR hexadrol OR isnacort OR isoptodex OR isoptomaxidex OR 'lokalison f' OR luxazone OR marvidione OR maxidex OR mediamethasone OR megacortin OR mephameson* OR metasolon* OR methazonion* OR millicorten OR millicortenol OR 'mk 125' OR mk125 OR nisomethasona OR novocort OR 'nsc 34521' OR nsc34521 OR opticorten OR opticortinol OR oradex*n* OR orgadrone OR policort OR posurdex OR prodexona OR prodexone OR sanamethasone OR spoloven OR triamcimetil OR visumethazone):ti,ab,kw
22	(melph*lan OR alkeran OR 'cb 3025' OR cb3025 OR 'levo sarcolysin' OR levofalan OR melfalan OR melphalon OR 'nsc 8806' OR nsc8806 OR 'phenylalanine 2037' OR 'phenylalanine mustard'):ti,ab,kw
23	(vincristine OR vincristin OR 'l 37231' OR l37231 OR 'vin cristine' OR vincrisul):ti,ab,kw
24	(cyclophosphamide OR 'b 518' OR b518 OR carloxan OR clafen OR cycloblastin* OR 'cyclofos amide' OR cyclofosfamid* OR cyclophosphamid* OR cyclophosphan* OR cyclostin OR cycloxan OR cyphos OR cytophosphan* OR cytoxan OR 'endocyclo phosphate' OR end*xan* OR genoxal OR 'mitoxan neosan' OR neosar OR noristan OR 'nsc 26271' OR 'nsc 2671' OR procytox OR procytoxide OR se*doxan):ti,ab,kw
25	(doxorubicin OR adriablastin OR adriablastin* AND adriacin OR adriamicin* OR adriblastin* OR caelyx OR doxil OR doxorubicine OR 'fi 106' OR fi106 OR lipodox OR myocet OR 'nsc 123127' OR nsc123127 OR rastocin OR resmycin OR 'rp 25253' OR rp25253 OR rubex OR sarcodoxome OR 'tlc d 99'):ti,ab,kw
26	(carmustine OR bcnu OR bicnu OR carmubis OR carmubris OR carmustin OR gliadel OR nitrumon OR 'nsc 409962'):ti,ab,kw
27	(prednisone OR ancortone OR biocortone OR colisone OR cortidelt OR 'de cortisyl' OR decortancyl OR de*ortin* OR dehydrocortisone OR delitisone OR deltacort*n* OR deltacortisone OR deltasone OR deltra OR 'di-adreson' OR diadreson OR en*orton* OR hostacortin OR insone OR meprison OR metacortandracin OR meticorten OR meticortine OR 'nsc 10023' OR nsc10023 OR orasone* OR paracort OR precort OR precortal OR prednisone* OR pronizone OR rectodelt OR ultracorten OR urtilone):ti,ab,kw

#	Search term
28	(prednisolone OR antisolon* OR aprednislon* OR benisolon* OR berisolon* OR caberdelta OR 'co hydeltra' OR codelcortone OR cortadelton* OR cortelinter OR cortisolone OR dacortin OR decortril OR dehydrocortex OR dehydrocortisol* OR dehydrohydrocortison* OR delcortol OR deltacortef OR deltacortenolo OR deltacortil OR deltacortoil OR deltaderm OR deltaglycortril OR deltahycortol OR deltahydrocortison* OR deltaophticor OR deltasolone OR deltastab OR deltidrosol OR deltisilone OR deltisolon* OR deltolasson* OR deltoson* OR dicortol OR domucortone OR encort*lon* OR glistelone OR hostacortin OR hydeltra OR hydeltrone OR hydrelta OR hydrocortancyl OR hydrocortidelt OR hydrodeltalone OR hydrodeltisone OR hydroretrocortin* OR inflanefran OR insolone OR keteocort OR leocortol OR mediasolone OR meprisolon* OR metacortalon* OR metacortandralon* OR metacortelone OR meticortelone OR metiderm OR morlone OR mydrapred OR nisolon OR nisolone OR 'nsc 9120' OR nsc9120 OR panafcortolone OR panafort OR paracortol OR phlogex OR precortalon OR precortancyl OR precortisyl OR predartrin* OR prednedome OR prednelan OR prednicoelin OR prednicort OR prednicortelone OR prednifor OR predniment OR predniretard OR prednis OR prednivet OR prednorsolon* OR predonine OR predorgasolon* OR prelone OR prenolone OR prezolon OR scherisolon OR serilone OR solone OR solupren* OR spiricort OR spolotane OR sterolone OR supercorti*ol OR taracortelone OR wysolone):ti,ab,kw
29	pomalidomide
30	(imnovid OR pomalyst OR “cc-4047” OR “cc 4047” OR cc4047):ti,ab,kw
31	panobinostat
32	(farydak OR “lbh-589” OR “lbh589” OR “lbh 589”):ti,ab,kw
33	carfilzomib
34	(kyprolis OR “pr-171” OR “pr171” OR “pr 171”):ti,ab,kw
35	daratumumab
36	(darzalex):ti,ab,kw
37	ixazomib
38	(ninlaro OR mln9708 OR “mln 9708” OR “mln-9708” OR (proteasome NEXT/1 inhibit*)):ti,ab,kw
39	elotuzumab
40	(empliciti OR HuLuc63 OR BMS-901608):ti,ab,kw
41	(#1 OR #2)
42	(#3 OR #4 OR #5 OR #6 OR #7 OR #8 OR #9 OR #10 OR #11 OR #12 OR #13 OR #14 OR #15 OR #16 OR #17 OR #18 OR #19 OR #20 OR #21 OR #22 OR #23 OR #24 OR #25 OR #26 OR #27 OR #28 OR #29 0 1 2 3 4 5 6 7 8 9 OR #40)
43	(#41 AND #42)
44	1q21
45	#43 AND #44

1.3 MEDLINE® In-Process

Database name	MEDLINE® In-Process
Search interface	http://www.ncbi.nlm.nih.gov/pubmed/
Date of search	8 April 2019
Time segment	None
Search filter	-

#	Search term
1	Search myeloma*
2	Search Bortezomib
3	Search Lenalidomide
4	Search Thalidomide
5	Search Bendamustine
6	Search Dexamethasone
7	Search Melphalan
8	Search Vincristine
9	Search Cyclophosphamide
10	Search Doxorubicin
11	Search Carmustine
12	Search Prednisone
13	Search Prednisolone
14	Search velcade
15	Search proteasome inhibitor
16	Search revlimid
17	Search treanda
18	Search cytoxan
19	Search endoxan
20	Search neosar
21	Search adriamycin
22	Search caelyx
23	Search doxil
24	Search gliadel
25	Search ancortone
26	Search encortone
27	Search pomalidomide
28	Search imnovid
29	Search pomalyst
30	Search panobinostat
31	Search farydak
32	Search carfilzomib
33	Search kyprolis
34	Search daratumumab
35	Search darzalex

#	Search term
36	Search ixazomib
37	Search ninlaro
38	Search elotuzumab
39	Search empliciti
40	Search (((((((((((((((((((((((((((((((((((((#2) OR #3) OR #4) OR #5) OR #6) OR #7) OR #8) OR #9) OR #10) OR #11) OR #12) OR #13) OR #14) OR #15) OR #16) OR #17) OR #18) OR #19) OR #20) OR #21) OR #22) OR #23) OR #24) OR #25) OR #26) OR #27) OR #28) OR #29) OR #30) OR #31) OR #32) OR #33) OR #34) OR #35) OR #36) OR #37) OR #38) OR #39
41	Search (#1) AND #40
42	Search 1q21
43	Search (#41) AND #42
44	Search (randomized controlled trial [pt] OR controlled clinical trial [pt] OR randomized [tiab] OR placebo [tiab] OR clinical trials as topic [mesh: noexp] OR randomly [tiab] OR trial [ti]) NOT (animals [mh] NOT humans [mh])
45	Search #43 AND #44
